# The burden of testicular cancer from 1990 to 2019 in the Middle East and North Africa region

**DOI:** 10.3389/fonc.2023.1276965

**Published:** 2023-12-22

**Authors:** Saeid Safiri, Kamaleddin Hassanzadeh, Sina Janbaz Alamdary, Seyed Ehsan Mousavi, Seyed Aria Nejadghaderi, Mark J. M. Sullman, Nima Naghdi-Sedeh, Ali-Asghar Kolahi

**Affiliations:** ^1^ Hematology and Oncology Research Center, Tabriz University of Medical Sciences, Tabriz, Iran; ^2^ Clinical Research Development Unit of Tabriz Valiasr Hospital, Tabriz University of Medical Sciences, Tabriz, Iran; ^3^ Department of Urology, Faculty of Medicine, Tabriz University of Medical Sciences, Tabriz, Iran; ^4^ Faculty of Medicine, Islamic Azad University Tabriz Branch, Tabriz, Iran; ^5^ Neurosciences Research Center, Aging Research Institute, Tabriz University of Medical Sciences, Tabriz, Iran; ^6^ Systematic Review and Meta-Analysis Expert Group (SRMEG), Universal Scientific Education and Research Network (USERN), Tehran, Iran; ^7^ Department of Life and Health Sciences, University of Nicosia, Nicosia, Cyprus; ^8^ Department of Social Sciences, University of Nicosia, Nicosia, Cyprus; ^9^ Social Determinants of Health Research Center, Shahid Beheshti University of Medical Sciences, Tehran, Iran

**Keywords:** testicular neoplasm, Middle East and North Africa, epidemiology, mortality, disability-adjusted life-years

## Abstract

**Background:**

The incidence rate of testicular cancer has risen in many countries during recent decades. This study aimed to outline the impact of testicular cancer on the Middle East and North Africa (MENA) region from 1990 to 2019, examining its burden by age group and according to the socio-demographic index (SDI).

**Methods:**

Data on the incidence, death, and disability-adjusted life-years (DALYs) due to testicular cancer were retrieved from the Global Burden of Disease study 2019. The counts and age-standardized rates (per 100,000) were reported, and all rates were accompanied by 95% uncertainty intervals (UIs).

**Results:**

In MENA, the age-standardized incidence rate of testicular cancer was 1.4 per 100,000 in 2019, showing a 244.0% increase since 1990. Similarly, the annual death rate, at 0.1, experienced a 2.6% rise during the same period. In 2019, testicular cancer accounted for 31.1 thousand DALYs, marking an age-standardized rate of 5.0, which was 2.8% higher than in 1990. The 1-4 age group exhibited the largest incidence rate in 2019. In addition, in both 1990 and 2019 the MENA/Global DALY ratio was higher than one in the 1-14 year age groups. During the period 1990 to 2019, the age-standardized DALY rate of testicular cancer steadily rose with higher SDI values, except for a decrease observed at an SDI of 0.8.

**Conclusion:**

Over the last thirty years, there has been a notable rise in the burden of testicular cancer in the MENA region.

## Introduction

Testicular cancer is a relatively rare malignancy, but its incidence has increased over the last few decades (1). In 2020, testicular cancer was the 20^th^ most common type of cancer, in terms of prevalence (2). Despite having a favorable long-term survival rate, patients with testicular cancer have a higher risk of complications (3). The risk factors for testicular cancer include genetic risk factors (especially mutations in the *KIT* and *RAS* genes), environmental exposures and underlying disorders (e.g., cryptorchidism, hypospadias and impaired spermatogenesis) (3, 4).

In 2020, testicular cancer accounted for 0.4% and 0.1% of the incident cases and deaths from all types of cancers, respectively (5). Between 1990 and 2019, there was a substantial global increase of about 46% in the age-standardized incidence rate of testicular cancer (6). Specifically in 2016, within the Middle East and North Africa (MENA) region, the age-standardized incidence rate for testicular cancer stood at 1.02 per 100,000, alongside a corresponding death rate of 0.25 per 100,000. Notably, this region ranked among the highest globally in terms of the burden measured by disability-adjusted life years (DALYs) (7).

Several previous reports have estimated the burden of different cancer types using information derived from the Global Burden of Disease (GBD) study (6, 8). For example, global reports have highlighted the burdens of urological cancers (9), bladder cancer (10), and prostate cancer (11). Utilizing GBD 2016 data, estimates of the global, regional and national burdens of testicular cancer have also been conducted (7). Furthermore, earlier investigations have detailed the worldwide incidence, mortality rates and trends associated with testicular cancer using the GLOBOCAN 2020 data (2, 12). However, no previous study has specifically focused on the MENA region. The MENA countries have large variations in the socioeconomic profile and health system capacities that may differ substantially from high-income countries (13). These disparities underscore the urgent need for comprehensive research into the burden of testicular cancer within this distinctive region. This study focuses on examining the burden of testicular cancer in the MENA region spanning the period 1990-2019, by age category and socio-demographic index (SDI).

## Methods

### Overview

The GBD Study 2019, overseen by the Institute of Health Metrics and Evaluation (IHME), measures the impact of 369 health conditions across the world (14). The GBD studies divide the world into 21 regions, with the MENA region consisting of 21 nations (Afghanistan, Algeria, Bahrain, Egypt, Iran (Islamic Republic of), Iraq, Jordan, Kuwait, Lebanon, Libya, Morocco, Oman, Palestine, Qatar, Saudi Arabia, Sudan, Syrian Arab Republic, Tunisia, Turkey, United Arab Emirates, and Yemen). The methods used in GBD 2019 have been reported in detail previously (14, 15). The information utilized in this article can be viewed using these links https://vizhub.healthdata.org/gbd-compare/ and http://ghdx.healthdata.org/gbd-results-tool. In the section below we provide a concise overview of the methodologies employed to generate the estimates reported in this article.

### Estimation framework

The International Classification of Diseases was used to define testicular cancer, including both version 10 (i.e., C62-C62.9, D29.2-D29.8, and D40.1-D40.8) and version 9 (i.e., 186-186.9, 222.0, 222.3, 236.4) (14). Data from the vital registration, verbal autopsy, and cancer registries were utilized to create the cause of death database. Mortality estimates were derived using mortality-to-incidence ratios (16). Mortality-to-incidence ratios were modeled using data from areas where mortality and incidence statistics for testicular cancer were both available for the same year. The mortality estimates obtained from the cancer registry and vital registration systems were used to inform the cause-of-death ensemble model (CODEm) (16). The CODEm generated mortality forecasts for locations where data was scarce or non-existent. The CODEm for testicular cancer included the following covariates: cumulative cigarette consumption, tobacco (cigarettes per capita), the healthcare access and quality index (HAQ), gender and age specific exposure to low consumption of fruit and vegetables, education (years per capita), lag distributed income per capita, and the SDI (16). Following this, CoDCorrect was used in an age-sex-state-year group to adjust the sum of the anticipated single-cause death estimations. The final estimates for mortality underwent division by the death-to-incidence ratio, yielding the incidence of testicular cancer (16). To calculate the 10-year prevalence of testicular cancer, the survival of each incidence cohort was modeled using the mortality-to-incidence as a scalar to consider where each country fell on a scale that ranged from the theoretical best to a theoretical worst survival rate. Impairment was assessed by dividing the total prevalence into four phases: 1. diagnosis or initial therapy; 2. remission; 3. metastatic; and 4. terminal (16). The diagnostic and main therapy phase included the period from the onset of symptoms until treatment had finished. The controlled phase started after primary treatment had finished and ceased when one of the following outcomes occurred: cure (defined as remaining free of recurrence and progression for 10 years); death from another cause; or progression into the metastatic phase. The metastatic phase is characterized by patients undergoing comprehensive treatment for metastatic illness, as established by SEER (Surveillance, Epidemiology, and End Results Program) averages (16). The last period was the term for the final month prior to dying. The different disability weights associated with each of the four sequelae are displayed in **Supplementary Table 1**. Patients that had been cured for over 10 years were separated into one of two different sequelae: diagnosis and primary therapy or the controlled phase of testicular cancer (16).

### Severity and years lived with disability

The years lived with disability (YLDs) were created by multiplying the individual disability weights with the sequelae specific prevalence rates. The disability weights of each cancer sequelae, along with their lay descriptions, have already been published (16). The YLD is a measure of a disease’s impact, with 1 YLD equaling one year of healthy life lost as a result of ill health or disability. Another crucial metric in assessing the disease burden is the disability-adjusted life year (DALY), calculated by combining the years of life lost (YLLs) and the YLDs.

### Compilation of results

All calculations were standardized by utilizing the GBD standard population (16), and 1000 iterations were executed at every phase of the estimation process to provide 95% uncertainty intervals (UIs). The 95% UIs were determined by the 25^th^ and 975^th^ values of the ordered estimates. The relationship between the burden of testicular cancer and SDI was examined using Smoothing Splines (17). SDI, ranging from 0 to 1, encompasses the fertility rate among individuals under 25 years old, the mean years of education for those over 15 years of age, and the lag-distributed mean income per capita (smoothed over the preceding 10 years). All visual representations were created using R statistical software (Version 3.5.2).

## Results

### The Middle East and North Africa region

In 2019, the MENA region was estimated to have had approximately 8.5 thousand (95% UI: 6.1 to 11.6) incident cases of testicular cancer, with an age-standardized incidence rate of 1.4 (1.0 to 1.8) per 100,000 – 244% higher (106.8 to 440.0) than that reported in 1990 (**Table 1**, **Supplementary Table 2**). Deaths attributed to testicular cancer totaled 520.0 (441.0 to 619.0) in 2019, with an age-standardized death rate of 0.1 (0.1 to 0.1) (**Table 1**, **Supplementary Table 3**). Moreover, the burden measured in DALYs due to testicular cancer amounted to 31.1 thousand (25.9 to 37.4), reflecting an age-standardized rate of 5.0 (4.2 to 5.9) (**Table 1**, **Supplementary Table 4**). Notably, no substantial alterations were observed in the age-standardized death and DALY rates of testicular cancer between 1990 and 2019.

**Table 1:** Incidence, deaths and DALYs due to testicular cancer in 2019 and the percentage change in the age-standardized rates during the period 1990-2019.

North Africa and Middle East	Incidence (95% UI)			Deaths (95% UI)			DALY (95% UI)		
	Counts (2019)	ASRs (2019)	Pcs in ASRs 1990-2019	Counts (2019)	ASRs (2019)	Pcs in ASRs 1990-2019	Counts (2019)	ASRs (2019)	Pcs in ASRs 1990-2019
	8496 (6144, 11622)	1.4 (1, 1.8)	244 (106.8, 440)	520 (441, 619)	0.1 (0.1, 0.1)	2.6 (-24, 35.8)	31123 (25882, 37443)	5 (4.2, 5.9)	2.8 (-25.9, 42.4)
Afghanistan	50 (26, 89)	0.1 (0.1, 0.2)	112.4 (5.2, 291.1)	17 (11, 25)	0.1 (0, 0.1)	73.9 (14.8, 157.1)	1076 (699, 1588)	2.8 (1.9, 4.2)	60.4 (-4.1, 155.4)
Algeria	312 (154, 567)	0.7 (0.4, 1.3)	179.6 (20.5, 542.7)	20 (14, 27)	0 (0, 0.1)	-10.5 (-39.9, 36.1)	1291 (938, 1738)	2.9 (2.1, 3.9)	-2.2 (-36.8, 50.1)
Bahrain	5 (3, 8)	0.4 (0.2, 0.7)	388.6 (123.3, 934)	0 (0, 0)	0 (0, 0)	54.9 (4.3, 131.5)	14 (10, 19)	1.2 (0.8, 1.6)	58.1 (9.9, 130.8)
Egypt	447 (210, 998)	0.4 (0.2, 0.9)	200.9 (22.7, 693.4)	44 (29, 66)	0 (0, 0.1)	14.5 (-24.5, 81.2)	2790 (1811, 4179)	2.7 (1.8, 4)	13 (-31.8, 87)
Iran	1704 (1067, 2400)	1.9 (1.2, 2.7)	495.7 (162.7, 1085.5)	91 (81, 103)	0.1 (0.1, 0.1)	176.2 (97.3, 280.5)	4940 (4114, 5763)	5.7 (4.7, 6.6)	176.1 (91.9, 294.2)
Iraq	382 (187, 798)	0.9 (0.5, 1.8)	187.4 (25, 573.1)	34 (23, 52)	0.1 (0.1, 0.1)	19.5 (-27.4, 105.7)	1975 (1315, 3047)	4.6 (3.2, 7)	14 (-32.8, 92.8)
Jordan	216 (111, 389)	1.8 (1, 3.2)	283.3 (78, 689.5)	12 (8, 16)	0.1 (0.1, 0.2)	1.6 (-34.8, 67.1)	735 (522, 1028)	6.3 (4.5, 8.5)	9.2 (-32.1, 80.9)
Kuwait	46 (20, 90)	1.1 (0.4, 2.3)	5.5 (-52.8, 162.7)	1 (1, 2)	0 (0, 0)	-57.4 (-68.8, -32.2)	80 (55, 130)	1.9 (1.3, 3)	-52.3 (-66.9, -20.5)
Lebanon	178 (91, 321)	3.3 (1.7, 5.9)	493.2 (150.8, 1202.8)	6 (4, 9)	0.1 (0.1, 0.2)	22.7 (-27.8, 108.1)	390 (249, 609)	7.2 (4.6, 11.1)	53 (-12.3, 163.1)
Libya	41 (20, 77)	0.6 (0.3, 1.1)	129 (-1.7, 421.8)	4 (2, 6)	0.1 (0, 0.1)	7 (-38.8, 94.5)	219 (144, 331)	3 (2, 4.4)	13.6 (-34.6, 97.5)
Morocco	106 (55, 208)	0.3 (0.2, 0.6)	173 (12.5, 536.8)	15 (10, 23)	0 (0, 0.1)	11.8 (-31.4, 84.4)	885 (555, 1367)	2.4 (1.5, 3.7)	14 (-34, 95.9)
Oman	35 (16, 62)	0.7 (0.3, 1.2)	351.8 (74.2, 974.4)	1 (1, 2)	0 (0, 0)	9 (-32.7, 82.5)	90 (56, 132)	1.8 (1.1, 2.4)	31.3 (-17.3, 114.2)
Palestine	13 (6, 25)	0.3 (0.2, 0.5)	330.4 (76.7, 900.1)	1 (1, 1)	0 (0, 0)	128 (44.4, 250.6)	67 (48, 88)	1.4 (1, 1.8)	102.8 (24.2, 227.2)
Qatar	37 (14, 71)	1.1 (0.4, 2)	288.5 (57.1, 794)	1 (1, 2)	0 (0, 0)	-11.7 (-44, 44.1)	80 (46, 125)	2.4 (1.5, 3.5)	9.3 (-32.6, 76.3)
Saudi Arabia	474 (235, 858)	1.3 (0.6, 2.4)	694.6 (211.4, 1674.9)	17 (10, 26)	0 (0, 0.1)	57.6 (-7.2, 167.3)	1160 (762, 1733)	2.8 (1.9, 4)	92.8 (17.5, 224.4)
Sudan	126 (59, 269)	0.3 (0.2, 0.6)	272.3 (30.1, 742.8)	20 (13, 29)	0.1 (0, 0.1)	89.2 (18.5, 209.6)	1239 (781, 1799)	2.9 (1.9, 4.2)	76.4 (-7.7, 219.5)
Syrian Arab Republic	74 (32, 165)	0.6 (0.3, 1.2)	250.3 (37.4, 822.5)	5 (3, 7)	0 (0, 0.1)	5.4 (-36.8, 77.2)	303 (201, 439)	2.3 (1.5, 3.3)	14.4 (-31.4, 90.4)
Tunisia	87 (44, 158)	0.8 (0.4, 1.5)	228 (27.1, 721.8)	4 (3, 6)	0 (0, 0.1)	3.4 (-37.7, 62.2)	261 (165, 387)	2.3 (1.5, 3.4)	15.7 (-31.4, 86.8)
Turkey	3946 (2169, 6678)	5 (2.6, 9)	288.2 (66.9, 767)	200 (144, 273)	0.2 (0.2, 0.3)	-21 (-50.1, 23.3)	11983 (8678, 16483)	14.5 (10.6, 20.3)	-12 (-44, 40)
United Arab Emirates	149 (68, 307)	1.3 (0.6, 2.5)	250.1 (47.1, 716.7)	13 (7, 27)	0.1 (0.1, 0.2)	70.3 (-1.2, 226.2)	768 (410, 1519)	6.4 (3.8, 11.3)	59.7 (-6.7, 206.9)
Yemen	60 (29, 122)	0.2 (0.1, 0.4)	226.7 (60.3, 572.8)	13 (8, 18)	0.1 (0, 0.1)	104.1 (31.2, 227)	743 (467, 1101)	2.4 (1.5, 3.5)	87.3 (16.3, 221.7)

DALY, disability-adjusted-life-years. (Generated from data available from http://ghdx.healthdata.org/gbd-results-tool).

### National level

In 2019, among the MENA nations, the age-standardized incidence rate for testicular cancer varied from 0.1 - 5.0 cases per 100,000. Turkey [5.0 (2.6 to 9.0)], Lebanon [3.3 (1.7 to 5.9)], and Iran [1.9 (1.2 to 2.7)] had the largest age-standardized incidence rates. Conversely, Afghanistan [0.1 (0.1 to 0.2)], Yemen [0.2 (0.1 to 0.4)], and Palestine [0.3 (0.2 to 0.5)] had the smallest rates (**Supplementary Table 2**).

In 2019, among the MENA countries, the age-standardized death rate for testicular cancer varied from 0.0 to 0.2 cases per 100,000 individuals. Turkey [0.2 (0.2 to 0.3)], the United Arab Emirates [0.1 (0.1 to 0.2)], and Jordan [0.1 (0.1 to 0.2)] had the largest age-standardized death rates, while the smallest were observed in Oman [0.0 (0.0 to 0.0)], Bahrain [0.0 (0.0 to 0.0)], and Palestine [0.0 (0.0 to 0.0)] (**Supplementary Table 3**).

In 2019, the age-standardized DALY rates for testicular cancer varied between 1.2 to 14.5 cases per 100,000 in the MENA countries. Turkey [14.5 (10.6 to 20.3)], Lebanon [7.2 (4.6 to 11.1)], and the United Arab Emirates [6.4 (3.8 to 11.3)] had the highest age-standardized DALY rates, while Bahrain [1.2 (0.8 to 1.6)], Palestine [1.4 (1.0 to 1.8)], and Oman [1.8 (1.1 to 2.4)] had the smallest (**Supplementary Table 4**).

The age-standardized incidence rate of testicular cancer was higher in 2019, than in 1990, in all MENA countries except for Kuwait and Libya. Saudi Arabia [694.6% (211.4 to 1674.9)], Iran [495.7% (162.7 to 1085.5)], and Lebanon [493.2% (150.8 to 1202.8)] had the biggest increases in the age-standardized incidence rate of testicular cancer (**Supplementary Table 2**).

The age-standardized mortality rate due to testicular cancer rose from 1990 to 2019 in six countries, while no substantial changes were observed in the other 15. Iran [176.2% (97.3 to 280.5)], Palestine [128.0% (44.4 to 250.6)], and Yemen [104.1% (31.2 to 227.0)] had the biggest increases in the age-standardized death rate (**Supplementary Table 3**).

The age-standardized DALY rate of testicular cancer increased from 1990 to 2019 in five of the MENA countries, while the DALY rate declined over this period in only one country. Iran [176.1% (91.9 to 294.2)], Palestine [102.8% (24.2 to 227.2)], and Saudi Arabia [92.8% (17.5 to 224.2)] had the biggest increases in the age-standardized DALY rate of testicular cancer, while only Kuwait [-52.3% (-66.9 to -20.5)] had a substantial decline in the DALY rate (**Supplementary Table 4**).

### Relationship with age

**Figure 1:**
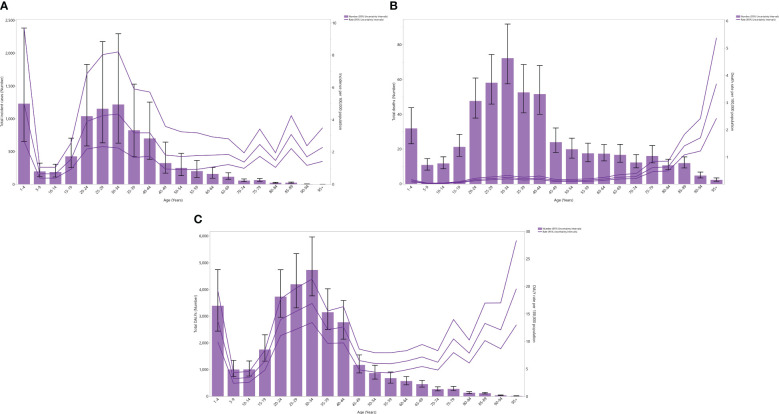
Number of incident cases and incidence rate **(A)**, number of death cases and death rate **(B)**, and the number of DALYs and DALY rate **(C)** for testicular cancer (per 100,000 population) in the Middle East and North Africa region, by age in 2019; Dotted and dashed lines indicate 95% upper and lower uncertainty intervals, respectively. DALY= disability-adjusted-life-years. (Generated from data available from http://ghdx.healthdata.org/gbd-results-tool).

In 2019, the regional incidence rate and incident cases of testicular cancer decreased dramatically up to the 5-9 age group, rose to those in the 30-34 age category, and then declined to those in the 95+ age category. In 2019, the 1-4, 30-34, and 25-29 age categories recorded the three highest incidence rates and incident cases of testicular cancer (**Figure 1A**).

The regional mortality rate in 2019 declined to the 5-9 age category, rose to the 40-44 age category, declined to the 45-49 age category, and then rose up to those aged 95+. Concurrently, in 2019, the death cases due to testicular cancer in the region decreased to the 5-9 age category, increased to the 30-34 age category, and then decreased to those aged 95+. Notably, in 2019, the 30-34, 25-29, and 35-39 age groups had the three highest incident cases of testicular cancer (**Figure 1B**).

In 2019, the regional DALY rate of testicular cancer declined up to the 5-9 age category, then rose to the 30-34 age category, declined to the 50-54 age group, and then rose to the oldest age group. Similarly, that same year, the total regional DALYs of testicular cancer declined to the 5-9 age category, rose to the 30-34 age category, and then declined to the oldest age category. In 2019, the 30-34, 25-29, and 20-24 age groups had the three highest regional DALYs of testicular cancer (**Figure 1C**).

**Figure 2:**
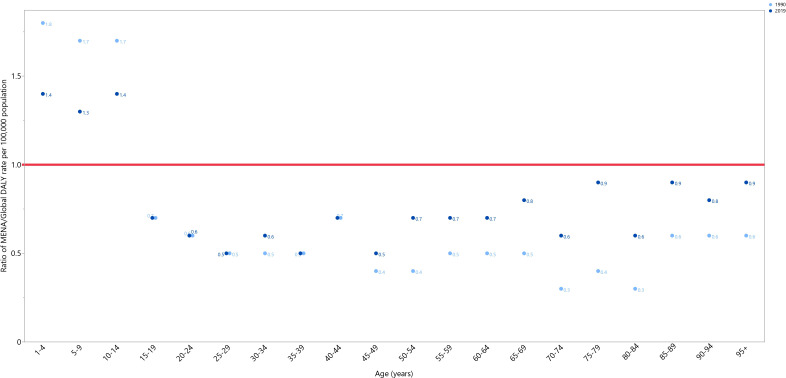
Ratio of the Middle East and North Africa region DALY rate to the global DALY rate of testicular cancer by age group, 1990–2019. DALY= disability-adjusted-life-years. (Generated from data available from http://ghdx.healthdata.org/gbd-results-tool).

In 1990 and 2019, the DALY rates of testicular cancer in MENA were below the global DALY rates (ratio of MENA/Global DALY rate < 1), except in individuals aged 1-14 years old. In 2019, the highest MENA/Global DALY rate ratios were seen in the 1-4, 10-14, and 5-9 age groups, while the lowest ratios were seen in the 25-29, 35-39, and 45-49 age categories. In comparison to 1990, in 2019 the MENA/Global DALY ratios were lower among those aged 1-14 years old, similar in the 15-29 and 35-44 age groups, and higher in all remaining age groups (**Figure 2**).

### Relationship with the Socio-demographic Index (SDI)

**Figure 3:**
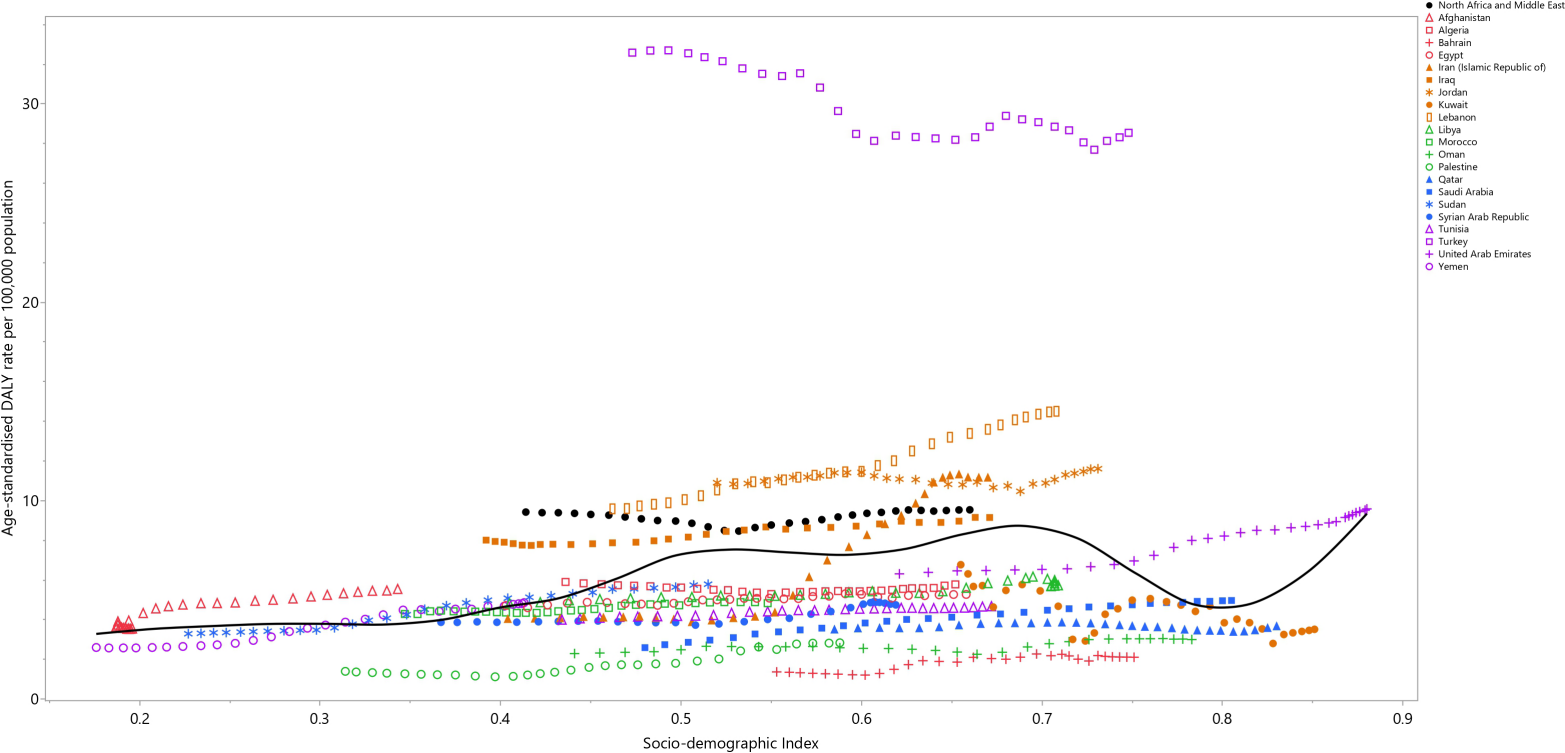
Age-standardized DALY rates of testicular cancer for the 21 MENA countries in 2019, by SDI; Expected values based on the Socio-demographic Index and disease rates in all locations are shown as the black line. Each point shows the observed age-standardized DALY rate for each country in 2019. DALY= disability-adjusted-life-years. SDI= Socio-demographic Index (Generated from data available from http://ghdx.healthdata.org/gbd-results-tool).

Between 1990 and 2019, the impact of testicular cancer steadily rose up to an SDI of around 0.7, then reduced to an SDI of 0.8, and then continued increasing to the highest SDI. Certain nations, like Iraq, Jordan, Lebanon, and Turkey, demonstrated burdens higher than expected. In contrast, Bahrain, Oman, Palestine, Qatar, the Syrian Arab Republic, and Tunisia displayed burdens that were lower than expected (**Figure 3**).

## Discussion

In this research, we utilized data from the GBD 2019 project to illustrate the impact of testicular cancer in the MENA region from 1990 to 2019. Our findings revealed a notable escalation in both the incidence and overall impact of testicular cancer over the previous thirty years. Moreover, the burden generally increased with advancing levels of socioeconomic status and was highest among the elderly and young adults.

Testicular cancer is a rare but highly curable malignancy, accounting for approximately 1% of all cancers in men (18). In the MENA region, we observed a surge of 244% in the age-standardized incidence rate of testicular cancer from 1990 to 2019. This increase is alarming, especially considering that testicular cancer is highly curable, if diagnosed early (19). The high incidence rates of testicular cancer in the MENA countries could be as a result of population ageing. It may also be due to various other factors, including changes in lifestyle and environmental factors (e.g., exposure to endocrine disruptors) (20).

It is essential to highlight the substantial divergence in testicular cancer incidence rates across MENA countries in 2019, with some countries having much lower incidence rates than others. For instance, Afghanistan, Yemen, and Palestine had the lowest incidence rates, whereas Turkey, Lebanon, and Iran had the highest. The observed differences in the incidence rates may stem from differences in the prevalence of several risk factors, such as undescended testes, testicular microlithiasis, genetics, and lifestyle factors. Additionally, a lack of awareness and inadequate screening programs in the region may result in delayed diagnosis and much worse outcomes. Performing testicular self-exams stands as a simple method for screening testicular cancer. However, several studies have shown a considerable lack of public awareness regarding testicular self-examination in the MENA countries (21, 22). This may lead to untreated testicular microlithiasis, which is thought to be associated with testicular cancer (23). In the MENA region, the feasibility and impact of screening programs versus educational initiatives can vary according to factors like the healthcare infrastructure and cultural considerations. In this context, both approaches could be valuable. Screening programs might require robust healthcare systems, while educational programs could enhance awareness. The choice might depend upon the specific health landscape of each individual country in MENA.

The impact of testicular cancer in the region is further highlighted by the fact that in 2019 it accounted for 31.1 thousand DALYs and 520 deaths. Improvements in access to health care services reduces the death rate from testicular cancer, so the associated DALY rate can be used as a measure of access to health services (24). The burden and incidence of testicular cancer vary across countries in the MENA region due to a variety of factors, including differences in the healthcare infrastructure, availability of diagnostic tools, and access to treatment. It is possible that the accessibility of chemotherapy and sonography will contribute to these differences. The availability of chemotherapy drugs and the capacity to administer them may also be limited in some of the MENA countries. This could result in delays in treatment, incomplete treatment, or the use of less effective treatment options, which could result in less desirable outcomes. Sonography or ultrasound imaging are commonly used diagnostic tools for testicular cancer (25). Sonography can be used to detect and evaluate testicular masses and to monitor treatment response. However, the availability of sonography may also vary across the MENA countries. Some countries may have limited access to sonography equipment, trained sonographers, or those able to interpret the imaging results, which could result in delays in diagnosis and treatment. It must be acknowledged that differences in the burden and incidence of testicular cancer may also be influenced by other factors, such as genetic predisposition, environmental factors, lifestyle factors, and access to healthcare in general. Genetic risk factors, especially mutations in the *KIT* and *RAS* genes, are also risk factors for testicular cancer (3, 4). To the best of our knowledge, there is no evidence to suggest that any of these genes are significantly higher in some MENA countries than in others. However, it is important to note that genetic predisposition to cancer can vary across populations, due to differences in genetic ancestry, environmental exposures, and lifestyle factors. Therefore, a comprehensive understanding of the factors contributing to differences in the impact of testicular cancer across the MENA nations requires further investigation and analysis.

Throughout the measurement period, there were no noteworthy fluctuations in the age-standardized death and DALY rates of testicular cancer. In both 1990 and 2019, within MENA, the DALY rate of testicular cancer surpassed the global DALY rate among those aged 1-14 years old. Conversely, the global DALY rate of testicular cancer reduced in the 1990s, possibly after the implementation of new therapeutic routines and approaches. The present study shows that the MENA countries have failed to address the increasing incidence of testicular cancer. This led to 31.1 thousand DALYs and 520 deaths in 2019.

There have been several advances in the treatment of testicular cancer since 1990. Some of the new therapeutic regimens and evidence-based approaches for treating testicular cancer include chemotherapy, surgery, radiotherapy, and immunotherapy. These treatments are available in most regions, including in the countries within MENA. However, the presence and accessibility of these treatment options may vary according to the country and healthcare system. Countries with a higher SDI included the new treatment strategies into their healthcare systems more quickly and more efficiently (26). Decreasing the DALY rates of testicular cancer in MENA, especially among those countries with lower SDIs, requires improvements in the healthcare infrastructure, accessibility to healthcare, and health education.

The age-specific incidence patterns of testicular cancer in the MENA countries typically showed a bimodal distribution, with incidence peaks in the 1-4 and 30-34 age groups. This pattern is similar to that observed in other regions of the world. The incidence rates of testicular cancer generally increase from childhood through to early adulthood, peaking notably in the third and fourth decades of life before exhibiting a gradual decline. The observed age-specific incidence patterns suggest that implementing early detection programs aimed at younger males could effectively alleviate the impact of testicular cancer in the region.

It is crucial to emphasize that our study relies on the GBD 2019 dataset, which, unfortunately, lacks a number of specific details such as ethnicity, histologic differences, and information about incidence stages or therapeutic approaches. The GBD’s limitations in providing such granular data impact our ability to delve deeper into these aspects of testicular cancer. Nevertheless, our research offers valuable insights into the overall trends of testicular cancer in the MENA region. We also acknowledge the importance of clarifying the population demographics in our study, particularly in countries like the Kingdom of Saudi Arabia and the Gulf States. However, it is essential to highlight the fact that the populations in these countries are not homogeneous, primarily due to the substantial presence of expatriates from Asian and Far East countries. This demographic heterogeneity can introduce variations in cultural backgrounds, lifestyles, and healthcare practices among the study participants (27). While our study has provided valuable insights within the scope of its objectives, we acknowledge the potential influence of these demographic factors on the generalizability of our findings. Future research endeavors may benefit from a more nuanced examination of the impact of expatriate populations on the health-related outcomes in countries within the region.

### Strengths and limitations

The use of GBD 2019 data facilitates a thorough assessment of testicular cancer’s impact in the MENA region, as it covers a wide range of countries and years. Furthermore, the use of a systematic approach to data analysis increases the validity and reliability of the findings. This study provides insights into the trends and patterns of testicular cancer in the region, which can inform healthcare policies and interventions to improve outcomes. However, it’s crucial to acknowledge the limitations inherent in this study that warrant consideration. The GBD 2019 database relies on modeling techniques to estimate any missing data, which may lead to bias in the results. In addition, the study is limited by the quality and availability of data, which may vary across countries and over time. Moreover, the analysis is limited to the parameters available in the GBD 2019 database, which may not capture all aspects of the burden of testicular cancer, such as the economic and social impacts on patients and their families. Finally, the study does not explore the underlying causes of the trends and patterns identified, which could provide additional insights into the impact of testicular cancer in the region.

## Conclusions

Over the past thirty years, there has been a large rise in the burden of testicular cancer within MENA. This trend is concerning, especially considering that testicular cancer is highly treatable. The observed differences in incidence rates and age-specific patterns suggest that targeted screening and awareness programs may be effective in lessening the impact of testicular cancer in the region. Additional research is necessary to identify the root causes of the increasing incidence rates and to develop effective prevention and early detection strategies.

## Author's note

This study is based on publicly available data and solely reflects the opinion of its authors, not that of the Institute for Health Metrics and Evaluation.

## Data availability statement

Publicly available datasets were analyzed in this study. This data can be found here: http://vizhub.healthdata.org/gbd-results/.

## Ethics statement

The present study was reviewed and approved by Ethics Committee of Shahid Beheshti University of Medical Sciences, Tehran, Iran (Ethics code: IR.SBMU.RETECH.REC.1402.046).

## Author contributions

SS: Conceptualization, Data curation, Formal Analysis, Methodology, Project administration, Resources, Supervision, Validation, Visualization, Writing – original draft, Writing – review & editing. KH: Writing – original draft, Writing – review & editing. SJ: Writing – original draft, Writing – review & editing. SM: Writing – original draft, Writing – review & editing. SN: Writing – original draft, Writing – review & editing. MS: Writing – original draft, Writing – review & editing. NN-S: Writing – original draft, Writing – review & editing. A-AK: Writing – original draft, Writing – review & editing.

## References

[1] Giona S. The epidemiology of testicular cancer. Brisbane, Australia: Exon Publications (2022) p. 107–16. 36343137

[2] Znaor A Skakkebaek NE Rajpert-De Meyts E Kuliš T Laversanne M Gurney J. Global patterns in testicular cancer incidence and mortality in 2020. Int J Cancer. (2022) 151(5):692–8. doi: 10.1002/ijc.33999 PMC12980092 35277970

[3] Rajpert-De Meyts E McGlynn KA Okamoto K Jewett MAS Bokemeyer C. Testicular germ cell tumours. Lancet (2016) 387(10029):1762–74. doi: 10.1016/S0140-6736(15)00991-5 26651223

[4] De Toni L Šabovic I Cosci I Ghezzi M Foresta C Garolla A. Testicular cancer: genes, environment, hormones. Front Endocrinology. (2019) 10. doi: 10.3389/fendo.2019.00408 PMC6626920 31338064

[5] Sung H Ferlay J Siegel RL Laversanne M Soerjomataram I Jemal A. Global cancer statistics 2020: GLOBOCAN estimates of incidence and mortality worldwide for 36 cancers in 185 countries. CA: A Cancer J Clin (2021) 71(3):209–49. doi: 10.3322/caac.21660 33538338

[6] Lin L Li Z Yan L Liu Y Yang H Li H. Global, regional, and national cancer incidence and death for 29 cancer groups in 2019 and trends analysis of the global cancer burden, 1990–2019. J Hematol Oncol (2021) 14(1):197. doi: 10.1186/s13045-021-01213-z 34809683 PMC8607714

[7] Pishgar F Haj-Mirzaian A Ebrahimi H Saeedi Moghaddam S Mohajer B Nowroozi MR. Global, regional and national burden of testicular cancer, 1990–2016: results from the Global Burden of Disease Study 2016. BJU Int (2019) 124(3):386–94. doi: 10.1111/bju.14771 30953597

[8] Global Burden of Disease Cancer C. Cancer incidence, mortality, years of life lost, years lived with disability, and disability-adjusted life years for 29 cancer groups from 2010 to 2019: A systematic analysis for the global burden of disease study 2019. JAMA Oncol (2022) 8(3):420–44. doi: 10.1001/jamaoncol.2021.6987 PMC8719276 34967848

[9] Zi H He S-H Leng X-Y Xu X-F Huang Q Weng H. Global, regional, and national burden of kidney, bladder, and prostate cancers and their attributable risk factors, 1990–2019. Military Med Res (2021) 8(1):60. doi: 10.1186/s40779-021-00354-z PMC8611255 34819142

[10] Safiri S Kolahi AA Naghavi M. Global, regional and national burden of bladder cancer and its attributable risk factors in 204 countries and territories, 1990-2019: a systematic analysis for the Global Burden of Disease study 2019. BMJ Glob Health (2021) 6(11). doi: 10.1136/bmjgh-2020-004128 PMC8634015 34844997

[11] Wang L Lu B He M Wang Y Wang Z Du L. Prostate cancer incidence and mortality: global status and temporal trends in 89 countries from 2000 to 2019. Front Public Health (2022) 10:811044. doi: 10.3389/fpubh.2022.811044 35252092 PMC8888523

[12] Huang J Chan SC Tin MS Liu X Lok VT-T Ngai CH. Worldwide distribution, risk factors, and temporal trends of testicular cancer incidence and mortality: A global analysis. Eur Urol Oncol (2022) 5(5):566–76. doi: 10.1016/j.euo.2022.06.009 35863988

[13] Lozano R Fullman N Mumford JE Knight M Barthelemy CM Abbafati C. Measuring universal health coverage based on an index of effective coverage of health services in 204 countries and territories, 1990–2019: a systematic analysis for the Global Burden of Disease Study 2019. Lancet (2020) 396(10258):1250–84. doi: 10.1016/S0140-6736(20)30750-9 PMC7562819 32861314

[14] Vos T Lim SS Abbafati C Abbas KM Abbasi M Abbasifard M. Global burden of 369 diseases and injuries in 204 countries and territories, 1990–2019: a systematic analysis for the Global Burden of Disease Study 2019. Lancet (2020) 396(10258):1204–22. doi: 10.1016/S0140-6736(20)30925-9 PMC7567026 33069326

[15] Murray CJL Aravkin AY Zheng P Abbafati C Abbas KM Abbasi-Kangevari M. Global burden of 87 risk factors in 204 countries and territories, 1990–2019: a systematic analysis for the Global Burden of Disease Study 2019. Lancet (2020) 396(10258):1223–49. doi: 10.1016/S0140-6736(20)30752-2 PMC7566194 33069327

[16] Wang H Abbas KM Abbasifard M Abbasi-Kangevari M Abbastabar H Abd-Allah F. Global age-sex-specific fertility, mortality, healthy life expectancy (HALE), and population estimates in 204 countries and territories, 1950–2019: a comprehensive demographic analysis for the Global Burden of Disease Study 2019. Lancet (2020) 396(10258):1160–203. doi: 10.1016/S0140-6736(20)30977-6 PMC7566045 33069325

[17] Wang Y. Smoothing splines: methods and applications. London, United Kingdom: Chapman and Hall/CRC (2019).

[18] Purdue MP Devesa SS Sigurdson AJ McGlynn KA. International patterns and trends in testis cancer incidence. Int J cancer. (2005) 115(5):822–7. doi: 10.1002/ijc.20931 15704170

[19] Jones RH Vasey PA. Part I: testicular cancer—management of early disease. Lancet Oncol (2003) 4(12):730–7. doi: 10.1016/S1470-2045(03)01278-6 14662429

[20] Irigaray P Newby J Clapp R Hardell L Howard V Montagnier L. Lifestyle-related factors and environmental agents causing cancer: an overview. Biomedicine Pharmacotherapy. (2007) 61(10):640–58. doi: 10.1016/j.biopha.2007.10.006 18055160

[21] Kuzgunbay B Yaycioglu O Soyupak B Kayis AA Ayan S Yavascaoglu I. (2013). Public awareness of testicular cancer and self-examination in Turkey: a multicenter study of Turkish Urooncology Society, in: Urologic Oncology: Seminars and Original Investigations, . Elsevier. 10.1016/j.urolonc.2011.01.020 21429771

[22] Ramim T Mousavi SQ Rosatmnia L Bazyar A Ghanbari V. Student knowledge of testicular cancer and self-examination in a medical sciences university in Iran. Basic Clin Cancer Res (2014) 6(3):7–11.

[23] Pedersen MR Rafaelsen SR Møller H Vedsted P Osther PJ. Testicular microlithiasis and testicular cancer: review of the literature. Int Urol nephrology. (2016) 48:1079–86. doi: 10.1007/s11255-016-1267-2 27007613

[24] Richards M Stockton D Babb P Coleman M. How many deaths have been avoided through improvements in cancer survival? Bmj (2000) 320(7239):895–8. doi: 10.1136/bmj.320.7239.895 PMC27327 10741993

[25] Thomas KL Jeong D Montilla-Soler J Feuerlein S. The role of diagnostic imaging in the primary testicular cancer: initial staging, response assessment and surveillance. Trans Andrology Urology. (2020) 9(Suppl 1):S3. doi: 10.21037/tau.2019.07.01 PMC6995841 32055480

[26] Greiman AK Rosoff JS Prasad SM. Association of Human Development Index with global bladder, kidney, prostate and testis cancer incidence and mortality. BJU Int (2017) 120(6):799–807. doi: 10.1111/bju.13875 28480994

[27] AlOmar RS Parslow RC Law GR. Development of two socioeconomic indices for Saudi Arabia. BMC Public Health (2018) 18(1):791. doi: 10.1186/s12889-018-5723-z 29940925 PMC6019717

