# Supplementary material for: The burden of testicular cancer from 1990 to 2019 in the Middle East and North Africa region
Source: Front Oncol. 2023 Dec 22;13:1276965. doi: 10.3389/fonc.2023.1276965 (PMC10767553; doi:10.3389/fonc.2023.1276965)
Supplement: Supplementary file 1 [file Table_1.docx]

| **Table S1: Sequelae for testicular cancer and the associated disability weights from the Global Burden of Disease 2019 Study** | | | |
| --- | --- | --- | --- |
| **Sequela** | **Lay description** | **Disability weight**  **(95% CI)** |  |
| Diagnosis and primary therapy phase of testicular cancer | This person has pain, nausea, fatigue, weight loss and high anxiety. | 0.288  (0.193-0.399) |  |
| Metastatic phase of testicular cancer | This person has severe pain, extreme fatigue, weight loss and high anxiety. | 0.451  (0.307-0.6) |  |
| Terminal phase of testicular cancer | This person has lost a lot of weight and regularly uses strong medication to avoid constant pain. The person has no appetite, feels nauseous, and needs to spend most of the day in bed. | 0.54  (0.377-0.687) |  |
| Controlled phase of testicular cancer | This person has a chronic disease that requires medication every day and causes some worry but minimal interference with daily activities. | 0.049  (0.031-0.072) |  |
