# Supplementary material for: The burden of testicular cancer from 1990 to 2019 in the Middle East and North Africa region
Source: Front Oncol. 2023 Dec 22;13:1276965. doi: 10.3389/fonc.2023.1276965 (PMC10767553; doi:10.3389/fonc.2023.1276965)
Supplement: Supplementary file 2 [file Table_2.docx]

| **Table S2: Incidence of testicular cancer in 1990 and 2019 and the percentage change in the age-standardised rates (ASRs) per 100,000 in the Middle East and North Africa region**  **(Generated from data available from http://ghdx.healthdata.org/gbd-results-tool)** | | | | | |
| --- | --- | --- | --- | --- | --- |
|  | **1990** | | **2019** | | **Percentage change in ASRs per 100,000** |
|  | **No (95% UI)** | **ASRs per 100,000 (95% UI)** | **No (95% UI)** | **ASRs per 100,000 (95% UI)** |  |
| **North Africa and Middle East** | **1476 (939 , 2818)** | **0.4 (0.3 , 0.7)** | **8496 (6144 , 11622)** | **1.4 (1 , 1.8)** | **244 (106.8 , 440)** |
| **Afghanistan** | **8 (4 , 19)** | **0.1 (0 , 0.1)** | **50 (26 , 89)** | **0.1 (0.1 , 0.2)** | **112.4 (5.2 , 291.1)** |
| **Algeria** | **70 (36 , 142)** | **0.3 (0.1 , 0.5)** | **312 (154 , 567)** | **0.7 (0.4 , 1.3)** | **179.6 (20.5 , 542.7)** |
| **Bahrain** | **0 (0 , 1)** | **0.1 (0 , 0.2)** | **5 (3 , 8)** | **0.4 (0.2 , 0.7)** | **388.6 (123.3 , 934)** |
| **Egypt** | **95 (43 , 280)** | **0.1 (0.1 , 0.4)** | **447 (210 , 998)** | **0.4 (0.2 , 0.9)** | **200.9 (22.7 , 693.4)** |
| **Iran** | **200 (90 , 452)** | **0.3 (0.2 , 0.6)** | **1704 (1067 , 2400)** | **1.9 (1.2 , 2.7)** | **495.7 (162.7 , 1085.5)** |
| **Iraq** | **55 (28 , 105)** | **0.3 (0.2 , 0.6)** | **382 (187 , 798)** | **0.9 (0.5 , 1.8)** | **187.4 (25 , 573.1)** |
| **Jordan** | **17 (9 , 30)** | **0.5 (0.3 , 0.8)** | **216 (111 , 389)** | **1.8 (1 , 3.2)** | **283.3 (78 , 689.5)** |
| **Kuwait** | **20 (8 , 47)** | **1.1 (0.5 , 2.4)** | **46 (20 , 90)** | **1.1 (0.4 , 2.3)** | **5.5 (-52.8 , 162.7)** |
| **Lebanon** | **19 (9 , 36)** | **0.6 (0.3 , 1)** | **178 (91 , 321)** | **3.3 (1.7 , 5.9)** | **493.2 (150.8 , 1202.8)** |
| **Libya** | **11 (6 , 22)** | **0.2 (0.1 , 0.4)** | **41 (20 , 77)** | **0.6 (0.3 , 1.1)** | **129 (-1.7 , 421.8)** |
| **Morocco** | **30 (17 , 56)** | **0.1 (0.1 , 0.2)** | **106 (55 , 208)** | **0.3 (0.2 , 0.6)** | **173 (12.5 , 536.8)** |
| **Oman** | **3 (1 , 7)** | **0.2 (0.1 , 0.3)** | **35 (16 , 62)** | **0.7 (0.3 , 1.2)** | **351.8 (74.2 , 974.4)** |
| **Palestine** | **2 (1 , 4)** | **0.1 (0 , 0.1)** | **13 (6 , 25)** | **0.3 (0.2 , 0.5)** | **330.4 (76.7 , 900.1)** |
| **Qatar** | **1 (1 , 3)** | **0.3 (0.1 , 0.6)** | **37 (14 , 71)** | **1.1 (0.4 , 2)** | **288.5 (57.1 , 794)** |
| **Saudi Arabia** | **30 (14 , 64)** | **0.2 (0.1 , 0.3)** | **474 (235 , 858)** | **1.3 (0.6 , 2.4)** | **694.6 (211.4 , 1674.9)** |
| **Sudan** | **20 (7 , 61)** | **0.1 (0 , 0.2)** | **126 (59 , 269)** | **0.3 (0.2 , 0.6)** | **272.3 (30.1 , 742.8)** |
| **Syrian Arab Republic** | **24 (11 , 52)** | **0.2 (0.1 , 0.3)** | **74 (32 , 165)** | **0.6 (0.3 , 1.2)** | **250.3 (37.4 , 822.5)** |
| **Tunisia** | **23 (11 , 54)** | **0.2 (0.1 , 0.5)** | **87 (44 , 158)** | **0.8 (0.4 , 1.5)** | **228 (27.1 , 721.8)** |
| **Turkey** | **829 (399 , 2025)** | **1.3 (0.7 , 2.9)** | **3946 (2169 , 6678)** | **5 (2.6 , 9)** | **288.2 (66.9 , 767)** |
| **United Arab Emirates** | **8 (4 , 14)** | **0.4 (0.2 , 0.6)** | **149 (68 , 307)** | **1.3 (0.6 , 2.5)** | **250.1 (47.1 , 716.7)** |
| **Yemen** | **9 (4 , 20)** | **0.1 (0 , 0.1)** | **60 (29 , 122)** | **0.2 (0.1 , 0.4)** | **226.7 (60.3 , 572.8)** |
