# Supplementary material for: The burden of testicular cancer from 1990 to 2019 in the Middle East and North Africa region
Source: Front Oncol. 2023 Dec 22;13:1276965. doi: 10.3389/fonc.2023.1276965 (PMC10767553; doi:10.3389/fonc.2023.1276965)
Supplement: Supplementary file 3 [file Table_3.docx]

| **Table S3: Deaths from testicular cancer in 1990 and 2019 and the percentage change in the age-standardised rates (ASRs) per 100,000 in the Middle East and North Africa region**  **(Generated from data available from http://ghdx.healthdata.org/gbd-results-tool)** | | | | | |
| --- | --- | --- | --- | --- | --- |
|  | **1990** | | **2019** | | **Percentage change in ASRs per 100,000** |
|  | **No (95% UI)** | **ASRs per 100,000 (95% UI)** | **No (95% UI)** | **ASRs per 100,000 (95% UI)** |  |
| **North Africa and Middle East** | **275 (205 , 379)** | **0.1 (0.1 , 0.1)** | **520 (441 , 619)** | **0.1 (0.1 , 0.1)** | **2.6 (-24 , 35.8)** |
| **Afghanistan** | **3 (2 , 5)** | **0 (0 , 0)** | **17 (11 , 25)** | **0.1 (0 , 0.1)** | **73.9 (14.8 , 157.1)** |
| **Algeria** | **12 (9 , 16)** | **0.1 (0 , 0.1)** | **20 (14 , 27)** | **0 (0 , 0.1)** | **-10.5 (-39.9 , 36.1)** |
| **Bahrain** | **0 (0 , 0)** | **0 (0 , 0)** | **0 (0 , 0)** | **0 (0 , 0)** | **54.9 (4.3 , 131.5)** |
| **Egypt** | **23 (16 , 42)** | **0 (0 , 0.1)** | **44 (29 , 66)** | **0 (0 , 0.1)** | **14.5 (-24.5 , 81.2)** |
| **Iran** | **19 (14 , 26)** | **0 (0 , 0.1)** | **91 (81 , 103)** | **0.1 (0.1 , 0.1)** | **176.2 (97.3 , 280.5)** |
| **Iraq** | **11 (8 , 16)** | **0.1 (0.1 , 0.1)** | **34 (23 , 52)** | **0.1 (0.1 , 0.1)** | **19.5 (-27.4 , 105.7)** |
| **Jordan** | **3 (2 , 5)** | **0.1 (0.1 , 0.2)** | **12 (8 , 16)** | **0.1 (0.1 , 0.2)** | **1.6 (-34.8 , 67.1)** |
| **Kuwait** | **1 (1 , 1)** | **0.1 (0.1 , 0.1)** | **1 (1 , 2)** | **0 (0 , 0)** | **-57.4 (-68.8 , -32.2)** |
| **Lebanon** | **3 (2 , 4)** | **0.1 (0.1 , 0.1)** | **6 (4 , 9)** | **0.1 (0.1 , 0.2)** | **22.7 (-27.8 , 108.1)** |
| **Libya** | **2 (1 , 2)** | **0 (0 , 0.1)** | **4 (2 , 6)** | **0.1 (0 , 0.1)** | **7 (-38.8 , 94.5)** |
| **Morocco** | **9 (7 , 13)** | **0 (0 , 0.1)** | **15 (10 , 23)** | **0 (0 , 0.1)** | **11.8 (-31.4 , 84.4)** |
| **Oman** | **0 (0 , 1)** | **0 (0 , 0)** | **1 (1 , 2)** | **0 (0 , 0)** | **9 (-32.7 , 82.5)** |
| **Palestine** | **0 (0 , 0)** | **0 (0 , 0)** | **1 (1 , 1)** | **0 (0 , 0)** | **128 (44.4 , 250.6)** |
| **Qatar** | **0 (0 , 0)** | **0 (0 , 0.1)** | **1 (1 , 2)** | **0 (0 , 0)** | **-11.7 (-44 , 44.1)** |
| **Saudi Arabia** | **4 (3 , 6)** | **0 (0 , 0)** | **17 (10 , 26)** | **0 (0 , 0.1)** | **57.6 (-7.2 , 167.3)** |
| **Sudan** | **6 (3 , 11)** | **0 (0 , 0)** | **20 (13 , 29)** | **0.1 (0 , 0.1)** | **89.2 (18.5 , 209.6)** |
| **Syrian Arab Republic** | **4 (3 , 6)** | **0 (0 , 0.1)** | **5 (3 , 7)** | **0 (0 , 0.1)** | **5.4 (-36.8 , 77.2)** |
| **Tunisia** | **3 (2 , 4)** | **0 (0 , 0)** | **4 (3 , 6)** | **0 (0 , 0.1)** | **3.4 (-37.7 , 62.2)** |
| **Turkey** | **167 (108 , 252)** | **0.3 (0.2 , 0.4)** | **200 (144 , 273)** | **0.2 (0.2 , 0.3)** | **-21 (-50.1 , 23.3)** |
| **United Arab Emirates** | **1 (1 , 2)** | **0.1 (0.1 , 0.1)** | **13 (7 , 27)** | **0.1 (0.1 , 0.2)** | **70.3 (-1.2 , 226.2)** |
| **Yemen** | **3 (2 , 5)** | **0 (0 , 0)** | **13 (8 , 18)** | **0.1 (0 , 0.1)** | **104.1 (31.2 , 227)** |
