# Supplementary material for: The burden of testicular cancer from 1990 to 2019 in the Middle East and North Africa region
Source: Front Oncol. 2023 Dec 22;13:1276965. doi: 10.3389/fonc.2023.1276965 (PMC10767553; doi:10.3389/fonc.2023.1276965)
Supplement: Supplementary file 4 [file Table_4.docx]

| **Table S4: DALYs due to testicular cancer in 1990 and 2019 and the percentage change in the age-standardised rates (ASRs) per 100,000 in the Middle East and North Africa region**  **(Generated from data available from http://ghdx.healthdata.org/gbd-results-tool)** | | | | | |
| --- | --- | --- | --- | --- | --- |
|  | **1990** | | **2019** | | **Percentage change in ASRs per 100,000** |
|  | **No (95% UI)** | **ASRs per 100,000 (95% UI)** | **No (95% UI)** | **ASRs per 100,000 (95% UI)** |  |
| **North Africa and Middle East** | **17451 (12629 , 26430)** | **4.8 (3.6 , 6.6)** | **31123 (25882 , 37443)** | **5 (4.2 , 5.9)** | **2.8 (-25.9 , 42.4)** |
| **Afghanistan** | **219 (116 , 385)** | **1.8 (1 , 2.6)** | **1076 (699 , 1588)** | **2.8 (1.9 , 4.2)** | **60.4 (-4.1 , 155.4)** |
| **Algeria** | **798 (585 , 1069)** | **3 (2.2 , 4)** | **1291 (938 , 1738)** | **2.9 (2.1 , 3.9)** | **-2.2 (-36.8 , 50.1)** |
| **Bahrain** | **4 (3 , 5)** | **0.7 (0.6 , 0.9)** | **14 (10 , 19)** | **1.2 (0.8 , 1.6)** | **58.1 (9.9 , 130.8)** |
| **Egypt** | **1526 (999 , 3169)** | **2.4 (1.7 , 4.5)** | **2790 (1811 , 4179)** | **2.7 (1.8 , 4)** | **13 (-31.8 , 87)** |
| **Iran** | **1210 (873 , 1709)** | **2.1 (1.5 , 2.9)** | **4940 (4114 , 5763)** | **5.7 (4.7 , 6.6)** | **176.1 (91.9 , 294.2)** |
| **Iraq** | **686 (483 , 977)** | **4.1 (2.7 , 6)** | **1975 (1315 , 3047)** | **4.6 (3.2 , 7)** | **14 (-32.8 , 92.8)** |
| **Jordan** | **198 (136 , 290)** | **5.7 (3.9 , 8.4)** | **735 (522 , 1028)** | **6.3 (4.5 , 8.5)** | **9.2 (-32.1 , 80.9)** |
| **Kuwait** | **69 (54 , 88)** | **4 (3.2 , 5)** | **80 (55 , 130)** | **1.9 (1.3 , 3)** | **-52.3 (-66.9 , -20.5)** |
| **Lebanon** | **151 (98 , 223)** | **4.7 (3.1 , 7)** | **390 (249 , 609)** | **7.2 (4.6 , 11.1)** | **53 (-12.3 , 163.1)** |
| **Libya** | **108 (73 , 156)** | **2.6 (1.7 , 3.8)** | **219 (144 , 331)** | **3 (2 , 4.4)** | **13.6 (-34.6 , 97.5)** |
| **Morocco** | **575 (408 , 822)** | **2.1 (1.5 , 3)** | **885 (555 , 1367)** | **2.4 (1.5 , 3.7)** | **14 (-34 , 95.9)** |
| **Oman** | **30 (19 , 44)** | **1.3 (0.8 , 2)** | **90 (56 , 132)** | **1.8 (1.1 , 2.4)** | **31.3 (-17.3 , 114.2)** |
| **Palestine** | **17 (11 , 26)** | **0.7 (0.5 , 1)** | **67 (48 , 88)** | **1.4 (1 , 1.8)** | **102.8 (24.2 , 227.2)** |
| **Qatar** | **12 (8 , 17)** | **2.2 (1.5 , 3.1)** | **80 (46 , 125)** | **2.4 (1.5 , 3.5)** | **9.3 (-32.6 , 76.3)** |
| **Saudi Arabia** | **257 (175 , 372)** | **1.5 (1 , 2.2)** | **1160 (762 , 1733)** | **2.8 (1.9 , 4)** | **92.8 (17.5 , 224.4)** |
| **Sudan** | **389 (185 , 828)** | **1.6 (0.9 , 2.9)** | **1239 (781 , 1799)** | **2.9 (1.9 , 4.2)** | **76.4 (-7.7 , 219.5)** |
| **Syrian Arab Republic** | **288 (186 , 425)** | **2 (1.3 , 2.9)** | **303 (201 , 439)** | **2.3 (1.5 , 3.3)** | **14.4 (-31.4 , 90.4)** |
| **Tunisia** | **187 (133 , 258)** | **2 (1.5 , 2.7)** | **261 (165 , 387)** | **2.3 (1.5 , 3.4)** | **15.7 (-31.4 , 86.8)** |
| **Turkey** | **10439 (6568 , 17093)** | **16.5 (10.6 , 25.9)** | **11983 (8678 , 16483)** | **14.5 (10.6 , 20.3)** | **-12 (-44 , 40)** |
| **United Arab Emirates** | **89 (62 , 132)** | **4 (2.9 , 5.7)** | **768 (410 , 1519)** | **6.4 (3.8 , 11.3)** | **59.7 (-6.7 , 206.9)** |
| **Yemen** | **188 (95 , 319)** | **1.3 (0.7 , 2.1)** | **743 (467 , 1101)** | **2.4 (1.5 , 3.5)** | **87.3 (16.3 , 221.7)** |
